# Thyroid hormone receptor **β** sumoylation is required for thyrotropin regulation and thyroid hormone production

**DOI:** 10.1172/jci.insight.149425

**Published:** 2021-08-23

**Authors:** Sujie Ke, Yan-Yun Liu, Rajendiran Karthikraj, Kurunthachalam Kannan, Jingjing Jiang, Kiyomi Abe, Anna Milanesi, Gregory A. Brent

**Affiliations:** 1 Division of Endocrinology, Diabetes and Metabolism, Department of Medicine, and Department of Physiology, David Geffen School of Medicine, UCLA and VA Greater Los Angeles Healthcare System, Los Angeles, California, USA.; 2 Department of Endocrinology, Union Hospital, Fujian Medical University, Fuzhou, Fujian, China.; 3 Wadsworth Center, New York State Department of Health, Albany, New York, USA.; 4 Department of Pediatrics and Department of Environmental Medicine, New York University School of Medicine, New York, New York, USA.; 5 Department of Endocrinology, Zhongshan Hospital, Fudan University, Shanghai, China.; 6 Department of Pediatrics, Keio University School of Medicine, Shinjuku-ku, Tokyo, Japan.; 7 Tokyo Saiseikai Central Hospital, Minato-ku, Tokyo, Japan.

**Keywords:** Endocrinology, Mouse models, Thyroid disease

## Abstract

Thyroid hormone receptor β (THRB) is posttranslationally modified by small ubiquitin-like modifier (SUMO). We generated a mouse model with a mutation that disrupted sumoylation at lysine 146 (K146Q) and resulted in desumoylated THRB as the predominant form in tissues. The THRB K146Q mutant mice had normal serum thyroxine (T4), markedly elevated serum thyrotropin-stimulating hormone (TSH; 81-fold above control), and enlargement of both the pituitary and the thyroid gland. The marked elevation in TSH, despite a normal serum T4, indicated blunted feedback regulation of TSH. The THRB K146Q mutation altered the recruitment of transcription factors to the *TSHβ* gene promoter, compared with WT, in hyperthyroidism and hypothyroidism. Thyroid hormone content (T4, T3, and rT3) in the thyroid gland of the THRB K146Q mice was 10-fold lower (per gram tissue) than control, despite normal TSH bioactivity. The expression of thyroglobulin and dual oxidase 2 genes in the thyroid was reduced and associated with modifications of cAMP response element–binding protein DNA binding and cofactor interactions in the presence of the desumoylated THRB. Therefore, thyroid hormone production had both TSH-dependent and TSH-independent components. We conclude that THRB sumoylation at K146 was required for normal TSH feedback regulation and TH synthesis in the thyroid gland, by a TSH-independent pathway.

## Introduction

Thyroid hormone (TH) exerts its biological and physiological actions primarily through interaction with the nuclear TH receptor (THR; refs. 1, 2). There are 2 THR genes, *THRA* and *THRB*, and each gene produces functional THR protein. THR α (THRA) is expressed predominantly in brain, heart, skeletal muscle, and adipose tissues. THR β (THRB) is expressed in liver, heart, and pituitary and mediates the regulation of cholesterol metabolism, as well as the feedback regulation of thyrotropin-stimulating hormone (TSH) in the pituitary. Mouse models with HA tags introduced into the THR genes to uniformly detect TR protein demonstrate that THRB1 is the predominant TR isoform in liver, and THRB2 protein, which contains an extended amino terminus, is the predominant form in the pituitary (3).

TSH is a glycoprotein that consists of 2 subunits, α and β. The α subunit is common to all of the pituitary glycoproteins. The β subunit (TSHβ) is directly regulated by TH/THRB2-mediated feedback mechanism (4, 5). The regulation of *TSHβ* gene expression involves multiple transcription factors (TFs) in addition to THRB, including GATA-binding protein 2 (GATA2) and Pit 1/POU1F1 (6–12). The role of these TFs, however, has largely been based on transient reporter expression assays rather than direct determination of in vivo binding to the *TSHβ* gene regulatory regions (11). Pit 1 is essential for normal anterior pituitary development, and individuals with a Pit 1 gene mutation in a region of the Pit 1 protein that disrupts function can develop anterior pituitary insufficiency, including central hypothyroidism and reduced growth hormone secretion (13). GATA2 is important for both stimulation and repression of *TSHβ* gene expression (9, 14). GATA-mediated transcription regulation has 2 modules: friend of GATA–dependent (FOG-dependent) and FOG-independent. FOG1 contains 8 zinc fingers and is the best characterized GATA factor. FOG1 heterodimerizes with GATA, recruits nucleosome remodeling factor and deacetylase, and then activates or represses transcription depending on the promoter context (15–18). FOG-dependent GATA action is involved in a range of diverse cellular processes (19, 20).

We previously showed that THRA and THRB were posttranslationally modified by small ubiquitin-like modifier (SUMO), important for TH/THR-mediated transcription (21). A number of TFs are sumoylated, including nuclear receptors, as well as kinases and phosphatases (22–25). SUMO modification involves the attachment of a SUMO, through covalently binding to a specific lysine motif in the target protein. The modification is reversible by SUMO peptidases that break the bond, called desumoylation. Sumoylation and desumoylation dynamically influence protein–protein interactions, protein structural conformation, and protein binding to DNA. Sumoylation is involved in a range of biological processes, including DNA replication, RNA transcription, cell proliferation and differentiation, metabolism, immune response, and neuronal function (26–29). Sumoylation defects have been associated with several diseases.

We previously reported that THRA has 2 sumoylation sites and THRB has 3 (21). Mutations at each of these sites reduce sumoylation in vitro and disrupt THR interaction with coactivators and corepressors. Reduction in THRA sumoylation was shown to impair Wnt signaling, resulting in reduced preadipocyte proliferation and interference with PPARγ-mediated adipogenesis (21, 30). In vitro studies showed that the sumoylation of THRs is important for receptor function and TH signal–mediated cellular functions. To determine the physiological role of THR sumoylation in vivo, we generated a mouse carrying a mutation at lysine 146 (K146) in THRB that is required for SUMO conjugation. In this mouse model, we demonstrate that the sumoylation of THRB at K146 was essential for the regulation of *TSHβ* gene expression and for normal TH production in the thyroid. Desumoylated THRB directly disrupted *TSHβ* gene regulation, as well as disrupting cAMP response element–binding protein–mediated (CREB-mediated) regulation of genes important for TH synthesis, including thyroglobulin (*Tg*) and dual oxidase 2 (*Duox2*).

## Results

### Generation of THRB sumoylation mutant mice.

**Figure 1:**
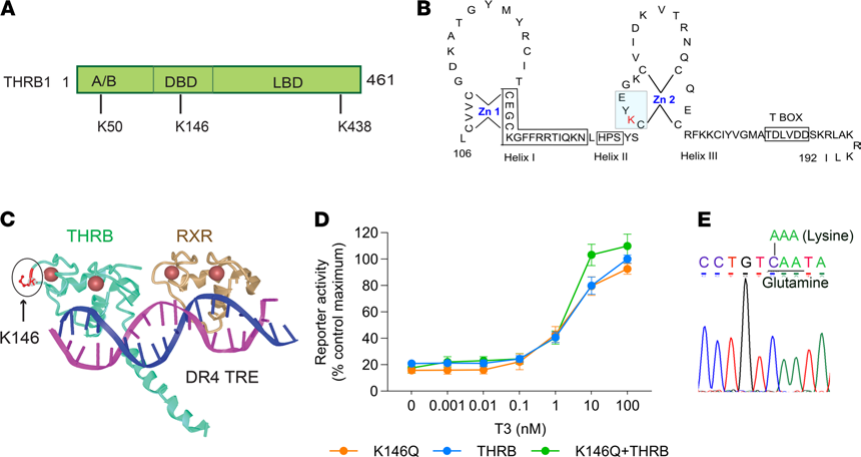
Location of sumoylation site K146 in the THRB1 protein and functional assay of the THRB1 K146Q mutant receptor. (**A**) Diagram of 3 sumoylation sites (K50, K146, K438) in the THRB1 protein indicating the amino terminus (A/B domain), DBD, and LBD. (**B**) Residue K146 is located within the Zn 2 of the THRB DBD (mutated K shown in red and sumoylation motif highlighted). (**C**) Ribbon diagram based on crystallographic data showing the THRB and RXR heterodimer bound to DNA DR4-TRE and the location of the mutated K146, outside of the region of direct DNA contact. (**D**) The THRB K146Q mutant was analyzed for its T3-mediated gene transcription in a reporter assay. The reporter contained 3 copies of the consensus DR4-TRE upstream of a luciferase gene. JEG3 cells were cotransfected with reporter and plasmids expressing THRB1 or THRB K146Q or a combination of THRB and K146Q. The amount of DNA in each transfection was kept constant. The luciferase activity was determined 12 hours after transfection using a multifunction plate reader. Results are presented as luciferase expression relative to the maximal THRB control transfection induction (shown as 100%). (**E**) The THRB point mutation in the mutant mice, from A to C, resulting in substitution of glutamine for lysine (K146Q), was confirmed by direct DNA sequencing. The THRB K146Q gene targeting strategy is shown in Supplemental Figure 1. Statistical analysis was performed for the reporter assay (**D**), using multiple paired *t* test in Prism statistical software. THRB, thyroid hormone receptor β; K146, lysine 146; DBD, DNA-binding domain; LBD, ligand-binding domain; Zn 2, second zinc finger; RXR, retinoid X receptor; DR4-TRE, direct repeat, 4-base pair gap, thyroid hormone response element.

THRB1 has 3 sumoylation sites located at K50, K146, and K438 (Figure 1A). K50 residue is located in the amino terminus (A/B domain); K438 residue is located in the ligand-binding domain; and K146 residue is located in the second zinc finger of the DNA-binding domain (DBD; Figure 1B). Based on crystallographic data, however, when THRB is DNA bound, K146 does not contact DNA (Figure 1C). Mutation of any of the 3 sites reduces THRB sumoylation in vitro, but the greatest functional disruption in our previous studies of adipocyte differentiation is from mutations at K146, suggesting a dominant role for this site (21, 30). We chose to substitute glycine for lysine at 146 (K146Q) to generate a sumoylation site mutation that would disrupt THR-SUMO conjugation without reducing ligand binding. We evaluated the influence of the K146Q mutation on the ability of THRB1 to mediate triiodothyronine-induced (T3-induced) transcription, utilizing a luciferase reporter with a consensus direct repeat with 4 base pair gap TH response element (DR4-TRE). The THRB K146Q mutant had the same ability as WT THRB to stimulate T3-mediated luciferase gene expression (Figure 1D), indicating that the THRB K146Q mutation had bound ligand and DNA and the mutations did not alter T3-stimulated gene transcription, as mediated by a DR4-TRE.

We generated a mouse model with a global THRB K146Q mutation, using traditional gene targeting techniques (Supplemental Figure 1; supplemental material available online with this article; https://doi.org/10.1172/jci.insight.149425DS1). The mutation was generated by a single nucleoside substitution, changing AAG (K) to CAG (Q). The *Thrb* isoforms, *Thrb1*, *Thrb2*, and *Thrb3*, are transcribed from the same gene by alternative start codons, such that all 3 THRB receptor isoforms were mutated simultaneously for the lysine (GenBank accession numbers *Thrb1* NM_001113417, *Thrb*2 NM_009380; *Thrb3* XM_011244745). The mutation position of nucleoside 1516 corresponds to the K146 residue in the THRB protein. The genotyping was based on the location in *Thrb1*, and the mutation was confirmed by high-resolution melting PCR and direct DNA sequencing (Figure 1E). The same gene mutation results in a substitution at K143 for THRB2 and at K115 for THRB3, and the mutations in the THRB2 and THRB3 receptor isoforms are located in the second zinc finger, as it is for THRB1. To simplify the presentation of the data, the THRB mutant mouse will be described as THRB K146Q, to represent the mutation in the analogous residue for all 3 THRB isoforms.

### Metabolic phenotype of THRB K146Q mice.

**Figure 2:**
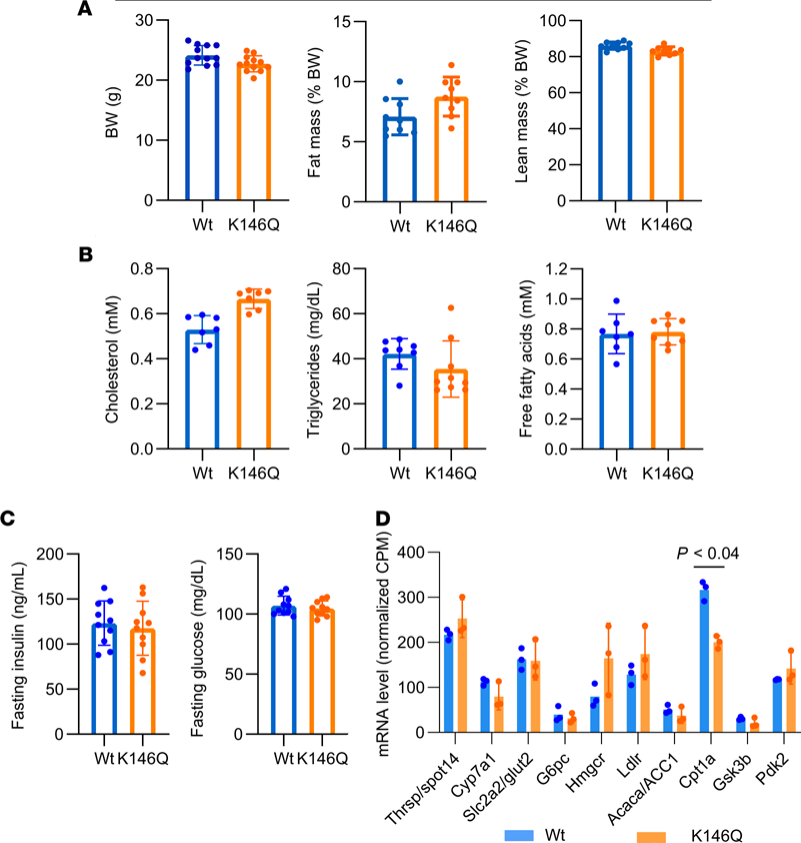
Metabolic phenotype of THRB K146Q mice. Mice (10 weeks old) were maintained at a normal light cycle on a regular chow diet and fasted for 6 hours before blood collection. Results are shown for WT (blue) and THRB K146Q mice (orange). Dots show individual values and bars indicate average ± SD. (**A**) BW is shown, and body composition, fat, and lean body mass are shown as a percentage of BW, as determined by Echo-MRI. (**B**) Serum concentrations of cholesterol, triglycerides, and free fatty acids. (**C**) Fasting glucose and serum insulin levels. (**D**) Transcriptome sequencing data show the mRNA expression level of known T3 target genes in the liver of THRB K146Q and WT mice. One-way ANOVA was used for statistical analysis, except transcriptome sequencing data (*P* < 0.04 is shown). Paired Student’s *t* test with normal distribution was used in statistical analysis. The statistical analysis for data shown in all panels was performed using Student’s *t* test for paired analysis. Thrsp/Spot14, thyroid hormone responsive protein; Cyp7a1, cytochrome P450 member 7a1; Slc2a2/Glut2, solute carrier family 2 member a2; G6pc, glucose-6-phosphatase catalytic subunit; Hmgcr, 3-hydroxy-3-methylglutaryl-CoA reductase; Ldlr, low density lipoprotein receptor; Acaca/ACC1, acetyl-CoA carboxylase 1; Cpt1a, carnitine palmitoyltransferase 1A; GSK3b, glycogen synthase kinase 3 β; Pdk2, pyruvate dehydrogenase kinase 2; THRB, thyroid hormone receptor β; K146, lysine 146; T3, triiodothyronine.

The body weight and body composition of the THRB K146Q mice (K146Q mice) was not different from WT mice (Figure 2A). Markers of TH action on metabolism, including fasting serum lipids (triglycerides, free fatty acid, and cholesterol), serum insulin, and glucose levels, showed no significant differences in K146Q mutant mice compared with WT mice (Figure 2, B and C). The levels of hepatic T3–responsive marker genes, important for lipid and glucose metabolism, were not significantly different in K146Q mutant mice compared with WT mice (Figure 2D), indicating no difference in the T3-mediated pathways that mediate lipolysis and lipogenesis.

### Thyroid status and changes in the pituitary and thyroid of THRB K146Q mice.

**Figure 3:**
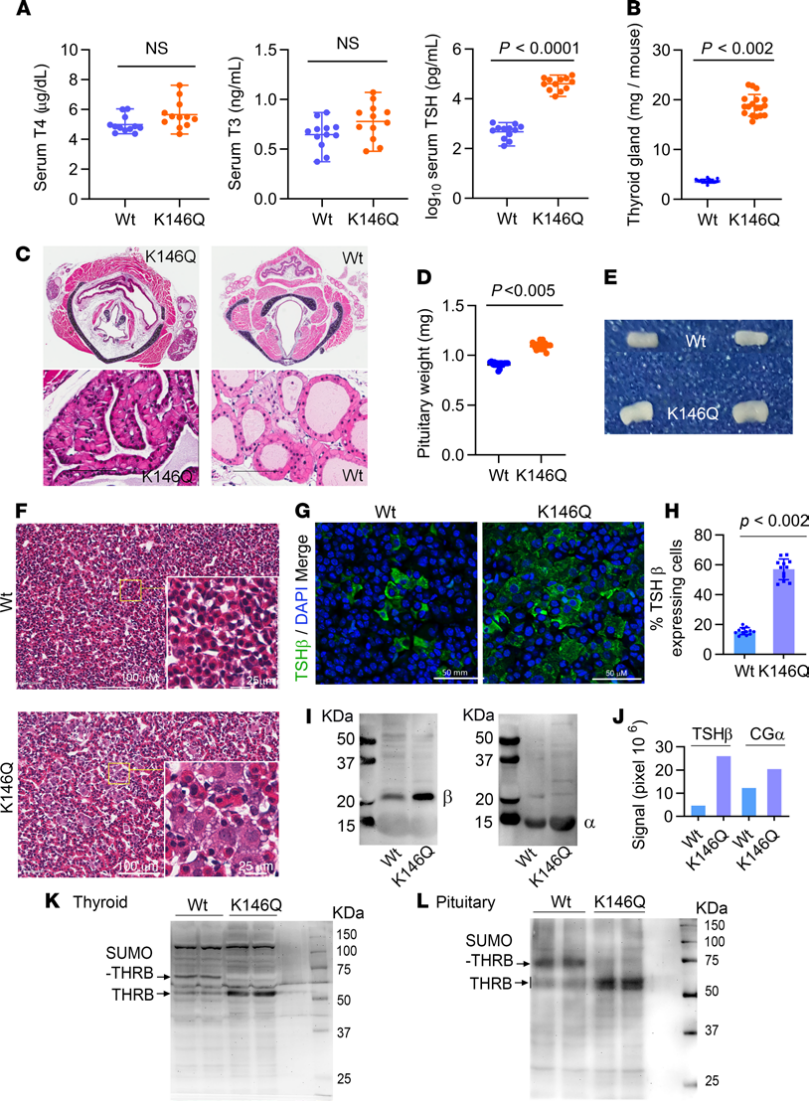
Thyroid status, thyroid gland, and pituitary findings in THRB K146Q mice. (**A**) Serum T4, T3, and TSH concentrations in WT and THRB K146Q mice (*n* = 13/ genotype) are shown as mean (±SD) and paired *t* test for statistical analysis. TSH is shown in log_10_ scale because of the wide differences in the levels in WT and K146Q mice. (**B**) Thyroid was rinsed with saline, patted dry, and weighed. The weight is shown as wet weight per mouse (*n* = 18/genotype). (**C**) Representative histology of thyroid gland stained with H&E. Transverse section of thyroid gland (top panel) and thyroid follicles (lower panel). (**D**) Dissected pituitaries were rinsed with saline, patted dry, and weighed, and values are shown as wet weight of pituitary from each mouse (*n* = 13/genotype). (**E**) Pituitaries are shown from WT and THRB K146Q mice. (**F**) Image of representative pituitary tissue histology with H&E stain from WT and THRB K146Q mutant mice. (**G**) Immunofluorescence staining for TSHβ (green) and for nuclei (DAPI blue). Frozen sections of the pituitaries were incubated with anti-TSHβ antibody at 1:50 dilution and conjugated with Alexa Fluor 488. (**H**) The TSHβ-expressing cells and total cell numbers were counted using green and blue filters. (**I**) Western blot detection of TSHβ and common glycoprotein α subunit (CGα) proteins. Pituitaries (*n* = 3) were lysed in RIPA buffer, and 30 μg of protein was loaded on an 8% SDS gel. Membranes were Ponceau S–stained (Supplemental Figure 2) prior to blot with anti-TSHβ and anti-CGα. (**J**) Quantification of TSHβ and CGα protein band in Western blot using LI-COR Image Studio Lite. (**K** and **L**) Western blot detection of THRB protein in the thyroid and pituitary. Protein (30 μg) was loaded onto a 10% SDS gel, transferred to a PVDF membrane, and blotted with anti-THRB antibody. The protein loading is shown (Supplemental Figure 3). Statistical analysis was performed using paired *t* test (**A**, **B**, **D**, and **H**). THRB, thyroid hormone receptor β; K146, lysine 146; TSH, thyrotropin-stimulating hormone, T4, thyroxine; T3, triiodothyronine.

Serum thyroxine (T4) and T3 levels in K146Q mice were not significantly different from control. The serum TSH level in the mutant mice, however, was markedly elevated and the mean level increased 81-fold compared with WT mice (Figure 3A). The thyroid in mutant mice was enlarged 5-fold by weight compared with WT mice (Figure 3B). Histological analysis of thyroid tissue showed hyperplastic thyroid follicles with scant colloid in K146Q mice compared with the normal follicles in WT mice (Figure 3C). Previously reported mouse models of resistance to thyroid hormone, due to mutation in the THRB gene (RTHβ), also show an elevated TSH and thyroid enlargement, but to a much smaller magnitude than seen in K146Q mice (31–35). The pituitary gland in K146Q mice was significantly enlarged — 20% — compared with control (WT 0.911 mg ± SD 0.028 and K146Q 1.093 mg ± SD 0.055, *n* = 13, *P* < 0.005; Figure 3, D and E). Pituitary hyperplasia and expansion of thyrotrophs were clearly visible in pituitary histology sections (Figure 3F). The marked increase in the number of thyrotrophs in the pituitary of K146Q mice was demonstrated by TSH antibody–stained pituitary sections (Figure 3G). In K146Q mice, 56% (SD ± 5.4%) of the pituitary cells stained positively for TSH, shown as green fluorescence, compared with 14% (SD ± 3.2%) in WT mice (*P* < 0.002; Figure 3H). The protein levels of common α subunit (α-glycoprotein) and *TSHβ* subunits were also increased, 1.65-fold and 5.6-fold respectively, in the pituitaries of mutant mice compared with WT (Figure 3, I and J, and Supplemental Figure 2). These data are consistent with the marked elevation of serum TSH in mutant mice. Mutation at the THRB sumoylation site K146 reduced TH feedback regulation of TSH, resulting in a marked elevation of TSH and promotion of thyroid growth, but did not increase TH production proportionate to the increase in serum TSH.

We investigated THRB protein expression in WT and K146Q mice to determine if there were significant changes in THRB protein content. The total THRB (combined sumoylated and desumoylated) in the thyroid and pituitary, detected by Western blot and quantitated by pixel density relative to protein loaded, was similar in WT and K146Q mutant mice (Figure 3, K and L, and Supplemental Figure 3). In the thyroid, the mean total THRB protein was 1.63% of the loaded protein in WT and 1.51% in K146Q mutant mice. In the pituitary, the mean total THRB was 2.06% of the loaded protein in WT mice and 1.98% in K146Q mutant. The total THRB content in thyroid and pituitary was similar (within less than 8%) in WT and K146Q mice. We then compared the proportion of sumoylated and desumoylated THRB. The desumoylated THRB molecular weight, as detected by Western blot, was 52–55 kDa. The addition of SUMO increased the molecular weight of protein with the predominant sumoylated THRB protein to 75 kDa in size (Figure 3, K and L). In WT mice, the mean sumoylated THRB was 44.7% of the total THRB protein in the thyroid and 78.8% of the total THRB in the pituitary. In K146Q mice, sumoylated THRB was not detectable by Western blot in the thyroid or pituitary; all THRB was desumoylated. There are 2 additional sumoylation sites in THRB that were not mutated, but based on this analysis, the K146 site, under the conditions we studied, was the only active sumoylation site in thyroid and pituitary.

### Regulation of TSH and TSH action.

**Figure 4:**
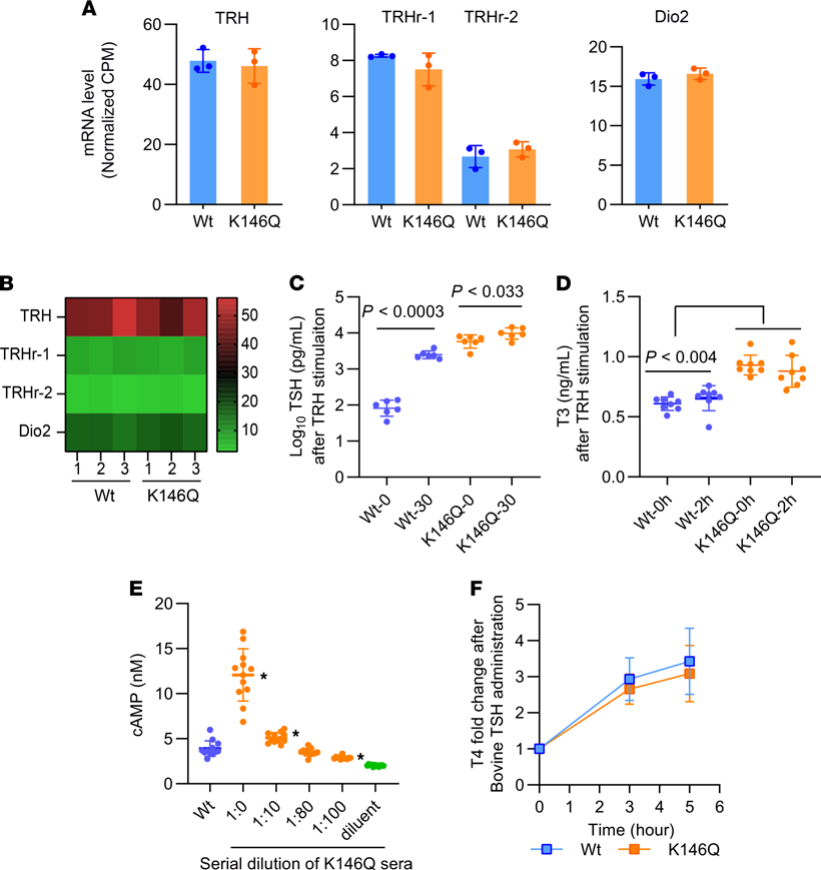
Exogenous TRH and TSH stimulation, hypothalamic gene expression, and TSH bioactivity in WT and THRB K146Q mice. (**A** and **B**) The mean (±SD) mRNA expression of hypothalamic TRH-associated genes by RNA-Seq are shown in bar figures and a heat map using 3 biological replicates (replicates 1, 2, 3) of WT and THRB K146Q mice. The scale of the intensity bar shows normalized CPM. The statistical analysis embedded in RNA-Seq data analysis software showed no significant differences. (**C** and **D**) Serum TSH and T3 in WT and THRB K146Q mice after TRH stimulation. A single dose of bovine TRH (5.0 μg/kg body weight) was injected (i.p.). Blood samples were collected at 0 and 30 minutes for TSH measurement and at 0 and 2 hours for T3 measurement. Individual values and mean ± SD are shown. Statistical analysis was performed with 2-way ANOVA to compare time points. (**E**) TSH bioactivity of serum from WT and THRB K146Q mice. CHO cells were transfected with TSHβ expression vector with/without β3-ADR expression vector. Sera of THRB K146Q mice were serially diluted with TSH-depleted sera. Mouse sera were added to the cells and incubated for 1 hour, and production of cAMP in CHO cells was determined. Statistical analysis was performed using 1-way ANOVA, *P < 0.01 compared with WT. (**F**) Mice were given a T3 injection (4 μg/100 g body weight/day) for 7 days to suppress TSH, and an i.p. injection of bovine TSH (200 mIU/100 g body weight) 20 hours after the last T3 injection. Serum T4 was analyzed at baseline and then 3 and 5 hours after bovine TSH injection. Statistical analysis was performed using paired Student’s *t* test. TRH, thyroid-releasing hormone; TRHr-1, TRH receptor-1; TRHr-2, TRH receptor-2; Dio2, Type 2 5′-deiodinase; THRB, thyroid hormone receptor β; K146, lysine 146; TSH, thyrotropin-stimulating hormone; TRH, thyroid-releasing hormone; T3, triiodothyronine; T4, thyroxine, CPM, counts per million.

TH feedback regulation occurs at both the hypothalamus, regulating thyroid-releasing hormone (TRH), and the pituitary, regulating TSH, which then stimulates the thyroid gland to produce TH. The hypothalamic mRNA levels of type 2 5′-deiodinase (*Dio2*), *TRH*, and TRH receptor (*TRH-R*) in K146Q mice were not different from the levels in WT mice (Figure 4, A and B). We additionally performed dynamic testing by administering bovine TRH and measuring the serum TSH and T3 in response. Serum TSH was measured at 30 minutes and 120 minutes after TRH injection and was stimulated in both mutant and WT mice, but to a greater level in WT mice, 19-fold, compared with 1.8-fold in mutant mice (Figure 4C). Serum T3 was measured at baseline and 2 hours after TRH injection, and there was no significant difference in the response between WT mice and K146Q mice (Figure 4D). Similar levels of *TRH* and *TRH-R* mRNAs in the mutant mouse hypothalamus, and preserved responsiveness of TSH to exogenous TRH, indicate that disordered TRH regulation was not likely to be a significant contributor to the abnormal phenotype.

The 5-fold enlargement of the thyroid gland in K146Q mice indicated that the elevated TSH was bioactive, with respect to thyroid growth, but the normal levels of serum T4 suggested reduced sensitivity to TSH action on hormone production. We directly evaluated TSH bioactivity by determining TSH-stimulated cAMP production in CHO cells transfected with mouse TSH-R. Serum was collected from WT and THRB K146Q mice. The TSH level of K146Q mice was 81-fold greater than that of WT mice; therefore, to compare the bioactivity of the same TSH concentration, serum from K146Q mice was diluted (0-fold, 10-fold, 80-fold, and 100-fold) with TSH-depleted mouse serum. The production of cAMP was 2.9-fold greater in CHO cells treated with undiluted serum from mutant mice, compared with treatment with serum from WT mice (*P* < 0.001). There was not a significant difference in cAMP production between WT serum and K146Q serum, with 10-fold and 80-fold dilution, respectively. At 100-fold dilution, cAMP production was reduced to 29% in K146Q mutant mice (*P* < 0.003) compared with WT serum (Figure 4E). The TSH bioactivity at equal concentrations was not significantly reduced in mutant mice compared with WT.

The observed reduced TSH action in stimulation of TH production could also have been the result of reduced TSH-R signaling. We analyzed short-term TSH-R function by dynamic testing, administering exogenous bovine TSH. Mice were rendered hyperthyroid by administration of T3 for 7 days to suppress endogenous T4 production from the thyroid. A single dose of bovine TSH was then administrated i.p. After exogenous TSH stimulation, serum T4 was compared with the baseline before stimulation (0 hour). Serum T4 levels increased 2.9-fold (*P* < 0.045) in WT mice after bovine TSH injection and 2.6-fold (*P* < 0.033) in K146Q mice, compared with the baseline level. There was no significant difference between WT and K146Q mice, indicating that TSH-R signaling was not altered. After prolonged stimulation, up to 5 hours, T4 production did not change significantly in either genotype (Figure 4F). These data indicate that the response to short-term TSH-R stimulation was normal in K146Q mice. Both in vivo and in vitro data indicate that the bioactivity of TSH and short-term TSH-R signaling was not different in mutant compared with WT mice.

### TSH regulation by T3.

**Figure 5:**
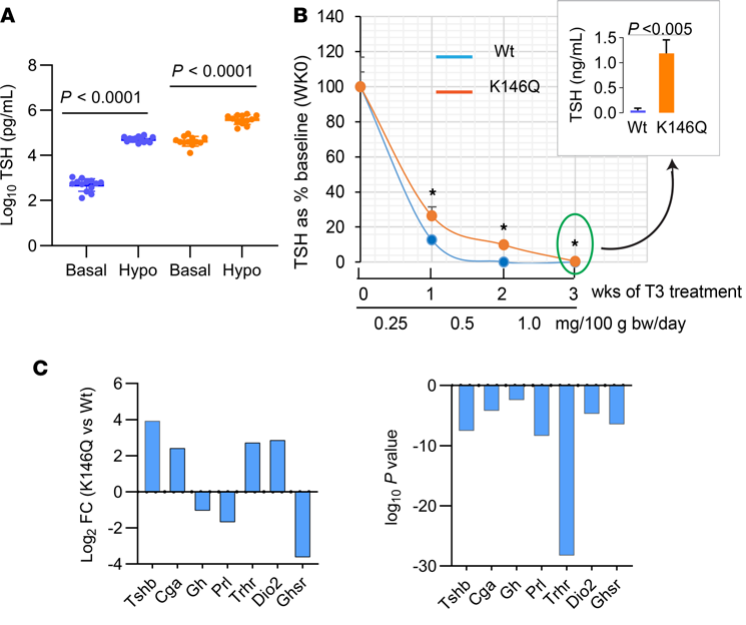
Response to exogenous T3 suppression in WT and THRB K146Q mice and pituitary mRNA profile. Mice (*n* = 10/genotype) were made to have hypothyroidism with a low iodine/PTU diet. Mice were then given T3 (i.p.) daily for a total of 3 weeks, with an increasing dose each week. Blood was collected at the end of each week, and TSH was assayed using ELISA kit (see details in Methods). (**A**) TSH levels before and after mice were rendered hypothyroid (hypo). TSH is shown on a log_10_ scale because of the wide differences in the TSH levels in WT and THRB K146Q mice. (**B**) TSH level shown as percentage baseline at week 0 of T3 treatment. Insert shows the actual TSH level at the end of 3 weeks of T3 suppression. Statistical analysis was performed using paired Student’s *t* test (**A** and **B**). (**C**) The anterior pituitary gene expression analyzed by RNA-Seq is shown as log_2_ fold change (logFC). *P* log_2_FC is shown in parallel graph for upregulated and downregulated genes. TSHb, TSHβ; CGα, common glycoprotein α subunit; Gh, growth hormone; Prl, Prolactin; Trhr, TRH receptor; Dio2, type 2 5′-deiodinase; Ghsr, growth hormone secretagogue receptor; T3, triiodothyronine; TSH, thyrotropin-stimulating hormone; PTU, propylthiouracil.

Reduced feedback response to T4 in the mutant mice could be the result of reduced Dio2 activity in the pituitary. In K146Q mice, however, *Dio2* mRNA was significantly increased (log fold change [FC] = 2.86, *P* < 1.97 × 10^10^) compared with WT mice. Pituitary feedback by TH was further assessed by graduated exogenous T3 suppression, as has been performed in evaluation of animals and humans with RTHβ (RTH caused by mutations of the THRB gene). Mice were rendered hypothyroid after 6 weeks on a low iodine and propylthiouracil (PTU) diet to stimulate TSH (Figure 5A and Table 1) and then treated with T3 for 3 weeks with a progressive increase in dose each week. At the end of the first week of T3 treatment (0.25 ng/100 g body weight/d), serum TSH of K146Q mice was reduced 76% (421 to 99 ng/mL) compared with baseline. Serum TSH in WT mice was reduced 87% (from 52.5 to 6.8 ng/mL), significantly lower than the levels in K146Q mice (*P* < 0.0015). Serum TSH in K146Q mice was further suppressed after 3 weeks of T3 treatment to 1.99 ng/mL, significantly higher than the level in WT mice (0.054 ng/mL; *P* < 0.001; Figure 5B). These data show that TSH in K146Q mice was responsive to T3 suppression, but the response was blunted, even at the highest doses of exogenous T3. The pituitary *TSHβ* mRNA content in K146Q mice was significantly increased by RNA-Seq analysis (log_2_FC = 4, *P* < 3 × 10^–8^) compared with WT mice, consistent with the marked increase in serum TSH (Figure 5C).

### The mechanism of K146Q and WT THRB regulation of TSHβ gene transcription.

**Figure 6:**
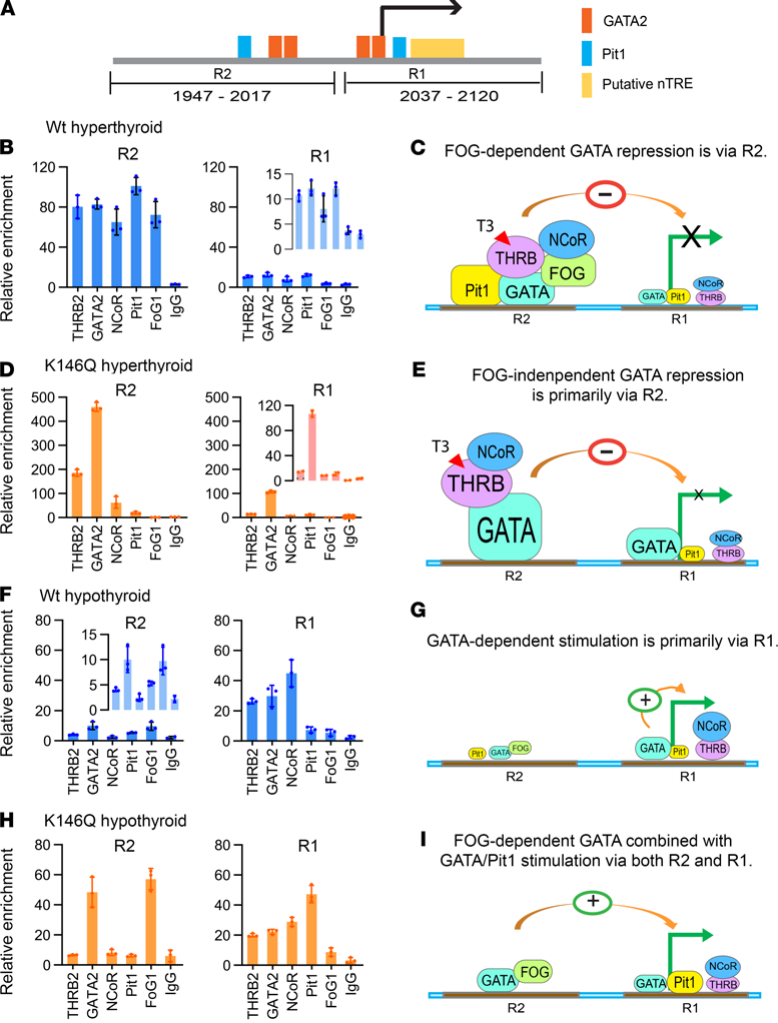
TF binding patterns to the *TSHβ* gene promoter in hyperthyroid and hypothyroid WT and THRB K146Q mice. Mice (*n* = 15/genotype) were made hyperthyroid with T3 injection or hypothyroid with low iodine/PTU diet. The pituitaries were removed and pooled for the ChIP assay to detect TFs’ (THRB2, GATA2, FOG1, NCoR) binding to DNA regions (R1 and R2). (**A**) Diagram of the *TSHβ* gene regulatory region mediated by T3. The numerical labeling of the regulatory region corresponds to mouse thyrotropin gene (GenBank accession number AH002109.2). The segment is divided into 2 regions (R1, R2) that bind TFs, with the R1 containing the TSS and negative thyroid hormone response element (nTRE). GATA, Pit 1, and THRB binding sites, identified by sequence, are shown. (**B** and **C**) ChIP analysis of TF binding in R2 and R1 of *TSHβ* gene promoter in hyperthyroid WT mice. Inset shows modified *y* axis scale with the same data. Graphic summary of T3-induced TF DNA binding in suppression of *TSHβ* gene transcription. The transcription start site is shown with a green arrow and the magnitude of TF binding by the size of the TF symbol. (**D** and **E**) ChIP analysis of TF binding to the *TSHβ* gene promoter in the pituitary of hyperthyroid K146Q mice. (**F** and **G**) ChIP analysis of TF binding to the *TSHβ* gene promoter in the pituitary of hypothyroid WT mice. (**H** and **I**) ChIP analysis of TF binding to the *TSHβ* gene promoter in the pituitary of hypothyroid K146Q mice. The chromatin assay (PCR) data represent DNA-bound TFs, and a 10% fraction of the chromatin lysate supernatant was used as the input control and labeled as “relative enrichment.” The statistical analysis described in the text utilized paired Student’s *t* test. THRB, thyroid hormone receptor β; K146, lysine 146; TSH, thyrotropin-stimulating hormone; GATA2, GATA-binding protein 2; T3, triiodothyronine; TFs, transcription factors; TSS, transcription start site; NCoR, nuclear receptor corepressor 1; PTU, propylthiouracil.

Sumoylation modifies protein–protein interactions of TFs required for gene transcription (36). We hypothesized that the desumoylated THRB altered the interaction with TFs required for regulating *TSHβ* gene transcription, including GATA2, Pit 1, THRB, and nuclear receptor corepressor 1 (NCoR) (6, 11, 12, 37). We utilized mouse pituitary tissue to directly determine the factors binding to the *TSHβ* gene regulatory region, by ChIP assay, and compared the pattern in K146Q mutant mice with that in WT mice. Mouse *TSHβ* gene 5′-flanking region (1847–2120 nt) contains multiple potential TREs, including those that bind GATA2 and Pit 1 and a putative negative TRE that binds THRB. To determine TF binding, the *TSHβ* gene promoter was divided into 2 regions (R1, R2) based on the distribution of the TFs and previously published data (Figure 6A). Because GATA2 is the key factor involved in *TSHβ* gene regulation, we first determined the GATA2 active binding site in pituitary tissue by ChIP assay. GATA2 binding was not detected in the further upstream segment of 1847–1947. Therefore, TF binding in the ChIP assays was studied only in the R1 and R2 segments.

Mice (WT and K146Q) were treated with T3 to suppress TSH, and the binding of TFs to the *TSHβ* gene regulatory region was evaluated by ChIP assay in pituitary tissue. WT mice treated with T3 had a low level of binding of TFs (THRB, NCoR1, GATA2, FOG1, and Pit 1) in the R1 region, whereas these TFs were highly bound to the R2 region (Figure 6, B and C). GATA2 recruited FOG1 to the promoter, indicating that FOG1-dependent GATA2 repression was involved in the inhibition of *TSHβ* gene. In K146Q mice, GATA2 binding to the R1 region was enriched 8-fold compared with WT mice in the same region (Figure 6, D and E). In the R2 region, enrichment of THRB and GATA2 was significantly increased 2-fold and 5-fold, respectively, compared with WT in the same region. However, FOG1 enrichment was not detected (Figure 6D). In summary, TH repression of *TSHβ* gene expression in WT mice was associated with FOG1-dependent GATA2 binding to R2, as well as binding of THRB, Pit 1, and NCoR1. In the THRB K146Q mutant mice, there was enhanced GATA binding to R1 and R2 but diminished FOG1 binding to R2, compared with binding in WT mice.

Mice (WT and THRB K146Q) were placed on a low iodine/PTU diet to examine ligand-independent activation of the *TSHβ* gene in hypothyroid mice. In WT hypothyroid mice, the enrichment of the TFs, THRB2, GATA2, and NCoR1, to the R1 region was increased 6.6-fold (*P* < 0.0003), 3.0-fold (*P* < 0.005), and 18-fold (*P* < 0.0001), respectively, compared with the R2 region (Figure 6, F and G). The enrichment data indicate that the R1 region, but not the R2 region, was involved in the activation of the *TSHβ* gene in WT mice. In THRB K146Q hypothyroid mice, the R1 region enrichment pattern for THRB2, GATA2, and NCoR1 was similar to that in WT mice, although with a significant increase in Pit 1 binding (3-fold greater, *P* < 0.05). In the R2 region, the enrichment of GATA2 and FOG1, but not the other factors, was increased significantly, 2.4-fold (*P* < 0.034) and 6.5-fold (*P* < 0.001), respectively (Figure 6, H and I). In summary, in WT mice, the activation of *TSHβ* gene transcription primarily involved factors binding to the R1 region. In contrast, in K146Q mice, *TSHβ* gene regulation involved binding to both the R1 and R2 regions. The TSH level in K146Q mice was high at baseline and increased further (2.6-fold) from weeks 2 to 6 with the low iodine/PTU hypothyroid diet. In comparison, serum TSH in WT mice on the hypothyroid diet increased 1.4-fold in the same period (Supplemental Figure 4). These data indicate that enhanced *TSHβ* gene expression in the hypothyroid K146Q mice was associated with increased TF binding to both the R1 and R2 regions of the *TSHβ* gene.

### Highly elevated serum TSH levels promote thyroid gland growth but do not enhance TH production.

**Figure 7:**
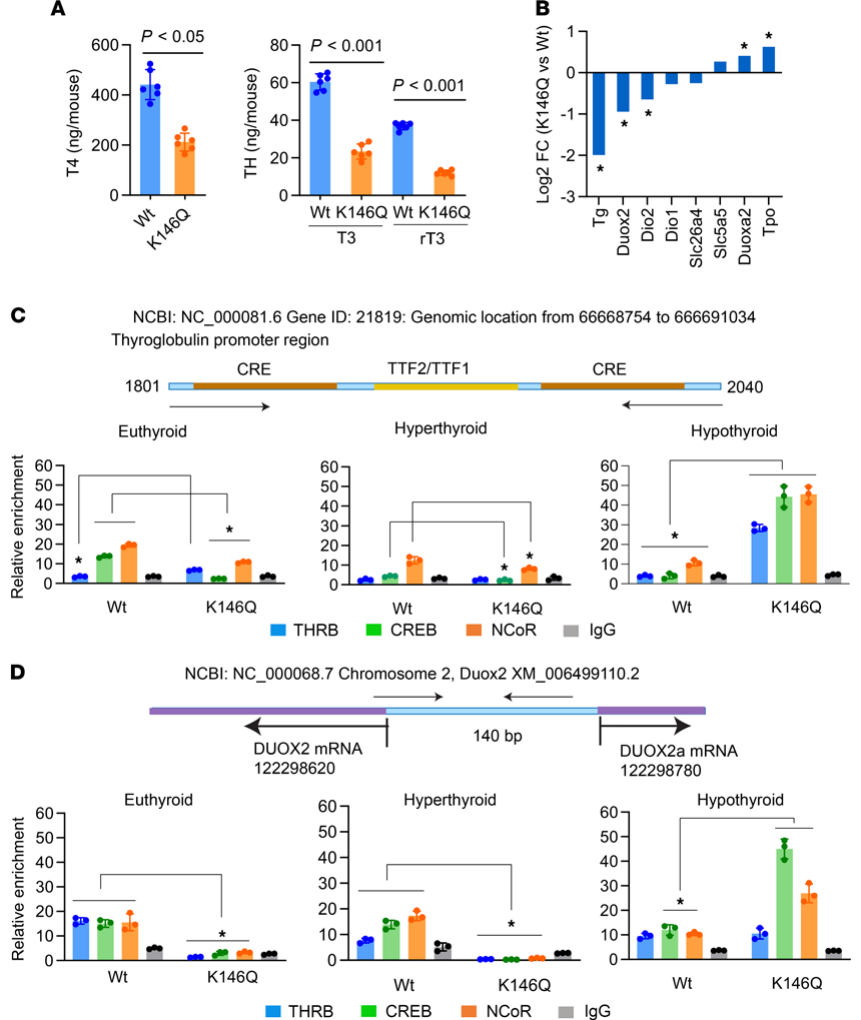
Characterization of thyroid hormone content, gene expression, and TF binding in *Tg* and *Duox2* gene regulator regions, in thyroid glands from THRB K146Q mutant and WT mice. (**A**) Thyroid hormone (T4, T3, rT3) levels in the thyroid gland were analyzed using liquid chromatography–tandem mass spectrometry. The data are normalized using average thyroid gland weight and total thyroid hormone content in WT and K146Q mouse and expressed per mouse (ng/mouse). The statistical analysis was performed with paired Student’s *t* test, and *P* values are shown, WT compared with K146Q mice. (**B**) RNA-Seq determination of the expression level of the genes relevant for thyroid hormone synthesis. (**C** and **D**) ChIP detection of TF enrichment to the Tg (**C**) and Duox2 (**D**) 5′ regulatory regions. Mice (*n* = 6/group) were in euthyroid, hypothyroid, or hyperthyroid conditions. The thyroid was dissected and pooled for ChIP assays using antibodies against THRB, CREB, and NCoR. The mapping of 5′ regulatory regions of the *Tg* and *Duox2* genes is shown above the quantitation of transcription factor binding. The horizontal arrows indicate the position of primers utilized for the ChIP assays. The ChIP data for various thyroid conditions are shown in the lower panels as the relative enrichment. Statistical analysis was performed using paired Student’s *t* test, comparison between WT and K146Q value, *P < 0.05 (**B**–**D**). THRB, thyroid hormone receptor β; K146, lysine 146; T3, triiodothyronine; T4, thyroxine; CREB, cAMP response element–binding protein; NCoR, nuclear receptor corepressor 1; *Tg*, thyroglobulin; *Duox2*, dual oxidase 2.

The mutant mice required a higher level of TH to suppress the TSH than WT mice, associated with reduced TH feedback inhibition of *TSHβ* transcription in K146Q mutant mice. The K146Q mutant mice displayed thyroid hyperplasia, but normal serum T4 levels, despite highly elevated serum TSH concentration. We investigated whether this was due to increased TH degradation in the thyroid, reduced TH synthesis, or both. We directly analyzed TH species (T4, T3, rT3, 3,3′T2, 3,5′T2, and 3′-T1) content in the thyroid and found that the levels of all were reduced, more than 10-fold in K146Q mutant thyroid, compared with the levels in the thyroid in WT mice (Figure 7A and Table 2). The expression of TH metabolism gene mRNA in the thyroid by RNA-Seq data showed no significant change in *Dio1* and reduced *Dio2* mRNA expression in mutant mice compared with WT. *Dio3* expression was increased in the mutant mice compared with WT, although its overall expression level was very low. There was no significant increase in thyroidal rT3 content, which would be expected if the elevated Dio3 were a factor reducing T4.

The key factors required for TH synthesis are the sodium iodide symporter (NIS), pendrin, thyroid peroxidase (TPO), DUOX2, and Tg. We found that RNA expression of *Tg* and *Duox2* was significantly downregulated, logFC 2 and logFC 0.95, respectively, in K146Q mutant mice compared with WT. *Tpo* was increased (logFC 0.63, *P* < 0.05), and mRNA of *NIS*, *sclc26a4* (pendrin), and *slc5a5* (MCT8) had no significant change (Figure 7B). DUOX2 is the primary dual oxidase to provide peroxide to TPO for iodination in thyroid. Thyroglobulin is the matrix substrate for TH synthesis and TH stores. The downregulation of these genes would be expected to impair TH synthesis. The transcriptional regulation of *Tg* and *Duox2* involves multiple factors; however, they are primarily regulated by cAMP through TSH/TSH-R signaling (38–41). Previous studies have shown that THRB interacts directly with CREB, as demonstrated by co-IP and ChIP assays, and influences CREB signaling (42–44). We hypothesized that the THRB K146 mutation may alter the normal pattern of THRB interaction with CREB and interfere with CREB-mediated *Tg* and *Duox2* gene regulation. To investigate, we performed ChIP assays using thyroid tissue collected from WT and mutant mice under euthyroid, hyperthyroid (T3-treated), and hypothyroid (low iodine/PTU diet) conditions. In mouse, the gene organization of *Tg* and *Duox2* is similar to human and rat (45–47), and CREB response elements (CREs) were identified in the promoter region of both genes (Figure 7, C and D).

In WT mice, the enrichment of CREB was 14.5%, and THRB was not bound to the *Tg* promoter, indicating that THRB was not normally involved in the regulation of *Tg* transcription in this region (Figure 7C). However, in K146Q mutant mice, THRB was recruited to the Tg promoter (*P* < 0.026 compared with WT mice), and CREB recruitment was lower than the IgG control. Compared with WT, the reduced CREB enrichment in mutant mice was consistent with the K146Q mutation interfering with CREB-mediated *Tg* transcription. Similarly, in hyperthyroid mice, CREB binding was also significantly reduced in K146Q mice. In hypothyroid mice, CREB and NCoR binding was highly enhanced in K146Q mutant mice compared with WT mice but was associated with marked blunting of *Tg* mRNA expression (Supplemental Figure 5). NCoR has been shown to inactivate CREB (48), and in hypothyroid conditions, the desumoylated THRB promotes NCoR and CREB binding. These findings are consistent with THRB K146Q mutant altering CREB binding and action and reducing TSH-mediated Tg gene expression.

In the *Duox2* promoter, THRB, CREB, and NCoR were enriched at a similar level in euthyroid mice (Figure 7D). In K146Q mutant mice, the enrichment of TFs to the promoter was not detectable after subtracting baseline IgG levels. The TF DNA binding pattern in *Duox2* promoter was similar to that seen on the Tg promoter; that is, K146Q mice showed reduced NCoR and CREB binding in hyperthyroidism and enhanced binding with hypothyroidism, compared with WT mice. The ChIP data showed a predominant pattern of reduced CREB binding on the Duox2 promoter in euthyroid and hyperthyroid conditions in K146Q mice and enhanced in hypothyroidism. Reduced expression of *Duox2* mRNA was seen in all conditions for K146Q mice compared with WT (Supplemental Figure 5). The THRB K146Q mutant modified CREB DNA binding and reduced CREB-mediated transcription of *Tg* and *Duox2*.

## Discussion

The K146Q mutant mouse model permitted us to study the physiological function of THRB-SUMO conjugation at K146 in vivo, without disrupting THRB binding to DNA. The mutation at residue K146 in THRB did not disrupt T3-induced gene expression of a DR4-TRE in transient transfection, but in vivo it impaired TSH regulation at the level of the pituitary and reduced TH synthesis. TH feedback suppression of TSH was diminished, resulting in a highly elevated serum TSH that was incompletely suppressed by exogenous T3 and thyrotroph hyperplasia in the pituitary. The highly elevated TSH promoted thyroid growth, but TSH action promoting TH production was diminished. By direct measurement, thyroidal TH (T4, T3, and rT3) levels were reduced in K146Q mice, calculated as THs per milligram thyroid tissue, as well as the total TH content per mouse thyroid (Table 2). ChIP assays of select genes important for TH synthesis showed that CREB-mediated expression of *Tg* and *Duox2* was disrupted by the THRB K146Q mutant. The mutation in THRB, tyrosine 147 to phenylalanine, also in the second zinc finger, was previously shown to disrupt THRB augmentation of PI3K signaling but did not impair TH signaling (49). Therefore, at least 2 residues contained in the THRB DNA binding domain, K146 and Y147, have regulatory roles distinct from mediating THRB binding to DNA.

We utilized pituitary tissue from WT and mutant mice to determine, by ChIP assay, the pattern of THRB and other TFs’ binding to the *TSHβ* gene promoter. Previous studies have mapped *TSHβ* regions important for TH-mediated feedback by the deletion and mutations of regulator regions in transient transfections, but the direct evaluation of binding of TFs in pituitary extract, even in WT mice, has been limited. The relative role of direct TR binding to DNA in TH-mediated downregulation of the *TSHβ* gene has varied by the model used, but in vivo models with mutation of the DBD support that TR binding is required (5). We showed that 2 regions (R1 and R2) in the *TSHβ* gene promoter were differentially involved in repression and stimulation of gene expression. We also demonstrated that the TFs, THRB2, GATA2, Pit 1, NCoR1, and FOG1, were involved in the transcription regulation of *TSHβ*. In hyperthyroid mice, suppression of *TSHβ* was mediated by the R2 region with FOG1-dependent GATA2 binding in WT mice. In contrast, FOG1-independent GATA2 binding was observed in K146Q mice, resulting in impaired feedback suppression of the *TSHβ* gene. When mice were made hypothyroid, binding of TFs was enriched to the R1 region, promoting *TSHβ* transcription in WT mice. In K146Q mice, however, TFs had bound to both R1 and R2, which was associated with excessive stimulation of the *TSHβ* gene and high levels of serum TSH.

The ChIP data show that the THRB K146Q mutation disrupted TH feedback regulation of TSH by altering the relative amount and configuration of TF DNA binding and interaction with regions R1 and R2. The level of THRB2 recruitment was similar in WT and THRB K146Q mice, so sumoylation did not directly influence THRB binding to DNA. Although there is no consensus TRE in the R2 region, THRB2 was largely enriched to this region in hyperthyroid mice, suggesting THRB2 was attached to GATA2 and required for TSH suppression, perhaps in a type 2 tethering role (50). NCoR1 was enriched to the R1 in hypothyroidism when *TSHβ* gene transcription was increased and enriched to R2 in hyperthyroidism for repression of *TSHβ* gene transcription. NCoR is a corepressor and recruited for inhibiting gene transcription. It was previously reported that mice with the NCoR ΔID mutation, which blocks interaction with THRB, have reduced serum TH, normal TSH levels, and increased T3 target gene expression in tissues (51), indicating an in vivo role of nuclear receptor corepressors in TH action. This phenotype suggests that NCoR1 is not involved in regulating THRB *TSHβ* gene expression but does modulate TH sensitivity in tissues (52). In our model, NCoR had bound to the *TSHβ* regulatory region with similar enrichment rate to that of WT THRB. The role of the interaction of NCoR and THRB on the *TSHβ* promoter needs to be further investigated.

GATA2-mediated transcription regulation has dual actions, FOG dependent and FOG independent. In FOG-dependent action, it recruits the nucleosome remodeling and deacetylase (NuRD) complex to remodeled chromosomal landscape, which may include DNA looping (16, 53). Although our current model did not address looping, FOG1 recruitment of NuRD is likely. Chromatin looping has been implicated to promote interaction between factors separated by more than 10 kb. Currently, there are no suitable tools available to precisely demonstrate looping. Pit 1 has been shown to be involved in TRH regulation of *TSHβ* expression (6, 54). In the pituitary nuclear extract ChIP assay, Pit 1 was also involved in transcription inhibition of *TSHβ* in WT mice but not in K146Q mice.

K146Q mice had thyroid hyperplasia, but the TH levels, including T4, T3, and rT3, in the thyroid were low. The main factors important for the TH synthetic pathway (NIS, TPO, Pendrin, DUOX2, and TG) were all stimulated by TSH, and most were regulated by TSH-activated cAMP signaling though CREB. The CREB binding sites have been studied and characterized in these genes using in vitro models (40, 47, 55–58). Our data show that K146Q selectively interfered with CREB-mediated Tg and Duox2 transcription but not all CREB signaling, such as the regulation of *Tpo* and *Slc5A5* expression. The extent to which desumoylated THRB interferes with CREB signaling may depend on the DNA sequence context for the CRE and the position in the promoter. In most cases, THRB does not directly contact the CRE, but rather it is tethered with CREB and disrupts CREB function (43, 44, 59, 60). Besides THRB interaction with CREB, Kruppel-like zinc finger TF GLI-similar (GLIS3) has recently been identified as a critical factor for the activation of *Slc26a4* (pendrin) and *Slc5A5* gene transcription. Deficiency in GLIS3 protein is linked to insufficient follicular cell proliferation, low TH production, and hypothyroidism in humans (61). In K146Q mice, GLIS3 was reduced (logFC = –0.6, *P* < 0.003). However, the mRNA levels of *Slc5A5* (logFC = 0.26, *P* < 0.11) and *Slc26a4* (logFC = –0.25, *P* < 0.72) were not changed in our THRB K146Q mice.

Local regulation of TH production, independent of TSH, has been shown to involve THRB (62) and, in another model, direct feedback by follicular Tg on genes important for TH synthesis (63). Thyrocyte-selective knockout of THRB resulted in reduced thyroid gland size and reduced TH synthesis. Thyroid hormone content, in contrast to the desumoylated model, was increased, likely due to a marked reduction in the TH transporters, Mct8 and Mct10, which are important for transporting TH out of the thyroid gland. Local feedback of Tg on TH synthesis has been linked to direct effects of Tg on reducing gene expression of genes important in TH synthesis (62).

In K146Q mice, highly elevated TSH with normal bioactivity did not stimulate TH production, but TH levels were reduced 10-fold. TH production is directly associated with TSH-stimulated thyroid TFs, paired box 8 and *SLC5a5* (coding for NIS), which in turn stimulates *Tg*, *Tpo*, and *Duox2* genes. THRB K146 selectively decreased CREB-mediated *Tg* and *Duox2* transcription in euthyroid and hypothyroid conditions and reduced cellular Tg protein. It is known that the shape and size of thyroid follicles are important for intracellular colloid content and thyroid function. Reduced TH production in K146Q mice may be due in part to the inappropriate morphological shape of the follicles from reduced intracellular colloid (64–66).

The robust phenotype, resulting from disruption of a single sumoylation site in THRB, indicates the likely importance of posttranslational THR modifications. Thyroid hormone signaling plays a central role in the balance of lipogenesis and lipolysis, but it also impacts glucose homeostasis. Our study showed that THRB sumoylation at K146 played a key role in TH regulation of TSH expression in the pituitary and TSH/TSHR-regulated TH production in thyroid. The presence of a regulatory system that could modulate central sensitivity to TH feedback and TH synthesis, perhaps from feeding or nutrient signals, may be relevant for normal physiology, as well as response to stress, systemic illnesses, and in metabolic diseases. It will be important to understand and characterize those factors that regulate THR sumoylation as well as the role of other THR posttranslational modifications.

## Methods

### Reporter assay.

Human choriocarcinoma JEG-3 cells (ATCC) express low levels of THRB. JEG-3 cells were maintained in Eagle’s minimum essential medium cell culture media supplemented with 10% FBS. Cells were plated to 24-well plates, 24 hours prior to transient transfection, and grown in serum-free media. Cells were transfected with plasmids using Attractene (QIAGEN) in Opti-MEM (Thermo Fisher Scientific). There were 6 replicates in each transfection. T3 was added to the medium at various concentrations, as indicated (Figure 1). Luciferase activity was determined 12 hours after transfection using a multifunction plate reader (Promega). The cells were cotransfected with the reporter and plasmids expressing THRB and/or THRB K146Q. The reporter vector carried 3 copies of a consensus DR4-TRE, upstream of the luciferase gene. For each transfection, an equal amount of DNA was used (0.2 μg reporter and 0.2 μg THRB or THRB K146Q). The reporter-only transfection included 0.2 μg of empty vector plasmid. In cotransfections, 0.1 μg of each THRB K146Q and THRB were used. The results were expressed as percentage of maximum luciferase activity of cells transfected with THRB (control).

### Generation of THRB sumoylation mutant mice.

The mutant mice were generated as whole-body knockin with a single nucleotide change at position 1516 (A to C), resulting in mutation of 1 THRB sumoylation site at lysine 146 (Supplemental Figure 1). The mutation was in a region common to all THRB isoforms (THRB1, THRB2, and THRB3). The mutant mice were generated on a C57BL/NTC background and then backcrossed to the same genetic background at least 3 times. All mutant mice used in the study were homozygous for the mutation. The WT mice were the same substrain as the mutant mice. Male mice were used in all studies, unless otherwise stated.

### Body composition analysis using Echo-MRI.

Mice were maintained on regular chow diet and a 12-hour light/12-hour dark cycle. The body composition of WT and THRB K146Q mice (*n* = 10/genotype) was determined by Echo-MRI at 9 to 10 weeks of age.

### Thyroid and pituitary histology and immunofluorescence staining.

Mice (*n* = 4/genotype) at 10 weeks of age were perfused by a transcardiac route with 4% paraformaldehyde. The thyroid and pituitary were then removed and underwent automated histology tissue processing including H&E stain and imaging in the UCLA Pathology Core Facility. The pituitary frozen sections were stained with anti-*TSHβ* antibody (LifeSpan Biosciences, LS-C334949) conjugated to Alexa Fluor 488 (Thermo Fisher Scientific, A32723), and the ProLong Gold antifade mountant with DAPI (Thermo Fisher Scientific, P36935) was applied. Each mouse had 3 sections evaluated with representative images shown.

### Western blot.

Three pituitaries from each genotype were pooled and lysed using RIPA buffer. The total lysate (30 μg/lane) was separated on an SDS-PAGE gel, and then a Western blot (WB) was performed using a standard protocol. We used the following antibodies: anti-*TSHβ* monoclonal antibody at 1:500 dilution for WB (Life Span Biosciences, LS-C334949) and anti-chorionic gonadotropin α at 1:1000 dilution for WB (Thermo Fisher Scientific, PA5-88517). THRB protein was assessed in the pituitary. Pituitaries were lysed in RIPA buffer with protease inhibitors and 20 mM *N*-ethylmaleimide. Two 10% SDS gels were prepared, 1 for WB and another for Coomassie blue staining. Protein (20 μg) was loaded 2 each lane, and gels were run in the same tank. Membrane was first blotted with anti-THRB antibody at 1:250 dilution (Abcam, ab104417). THRB protein was assessed in the thyroid. Crude lysate (30 μg) of thyroid gland from each mouse was loaded on a 10% SDS-PAGE gel. Detection of THRB and sumoylated THRB in the thyroid gland was carried out using ab-THRB1 (Abcam, ab180612) at 1:500 dilution.

### Hormones, lipids, and insulin.

Serum TSH, T3, and T4 levels were determined using the Rat Thyroid Hormone ELISA Kit (Millipore catalog RTHYMAG-30K) by Luminex 200 Bioanalyzer and MILLIPLEX analyst v5.1 software. Serum fasting free fatty acid, total cholesterol, and triglycerides were analyzed by ELISA Kit (Cayman Chemical). Serum insulin was measured by ELISA Kit (Invitrogen, Thermo Fisher Scientific).

### TSH suppression by exogenous T3.

Mice at 9 weeks of age (WT and THRB K146Q sumoylation mutant) were divided into 2 groups: low iodine plus 0.15% PTU diet for 6 weeks and control. Serum was collected for measurement of T4, T3, and TSH. Mice were then given T3 (MilliporeSigma) by i.p. injection for 3 weeks, with an increasing dose each week, 25 μg, 50 μg, and 100 μg/100 g body weight, per day. Serum was collected for the measurement of T4, T3, and TSH levels at the end of each week.

### Assay of TSH stimulation of T4 production.

Mice were given a T3 injection (4 μg/100 g body weight/d, i.p.) for 7 days to suppress endogenous TSH. Serum TSH and T4 were analyzed before and 20 hours after the final injection. Mice were then given bovine TSH (MilliporeSigma) 200 mIU/100 g body weight (i.p.) to directly stimulate the thyroid gland. The TSH and T4 levels were analyzed at 3 hours and 5 hours after the injection of bovine TSH.

### TRH stimulation of TSH.

WT and THRB K146Q mice, 9 weeks of age, were given a single i.p. dose of bovine TRH (5.0 μg/kg body weight). After injection, serum samples were collected at 30 minutes for TSH and at 2 hours for measurement of T4 and T3. TRH was purchased from MilliporeSigma.

### TSH bioactivity test with cAMP ELISA.

CHO cells were maintained with Hanks’ F-12K medium supplemented with 10% FBS and an antibiotic mixture of penicillin and streptomycin (ATCC). CHO cells (2 million cells/reaction) were transfected with a vector expressing mouse TSH-R and/or adrenergic receptor β 3 using a Nucleofector 4D device and cell type–specific reagent (Lonza). After the electroporation, cells were plated to a 24-well plate (~2 × 10^5^ cells/well). The assay was based on a previously described method (67) with modifications. In brief, on the day of testing, mouse serum was collected from whole blood centrifuged at 8000*g* for 3.3 minutes at 4°C and kept on ice. The assay buffer was freshly made and composed of Krebs-Ringer HEPES buffer (pH 7.4) and 8 mM glucose with 0.5 g/L BSA (KRPH-DG-BSA) and filtered through a 0.2 μm membrane. Cells were washed twice with 300 μL KRPH-DG-BSA, then incubated in the same buffer for 30 minutes in culture incubator. Medium was then removed and fresh KRPH-DG-BSA buffer containing 30 μL of mouse serum and 25 μM rolipram (Calbiochem), a cAMP phosphodiesterase inhibitor, was added and then incubated for 1 hour. In the positive control, mouse serum was replaced by 10 μM forskolin (MilliporeSigma). At the end of the incubation, cells were washed twice with 200 μL cold PBS and then lysed in 100 μL lysis buffer. A cAMP ELISA was performed following kit instructions (Cell Signaling Technology). Because of the very high TSH level in mutant mice, the serum of mutant mice used in the assay was diluted with TSH-depleted serum at ratios of 1:10 and 1:100.

### Preparation of TSH-depleted mouse serum.

TSH-depleted serum was prepared by treatment of mice with levothyroxine 5 μg/100 g body weight for 10 days, which suppresses endogenous TSH production. Blood was drawn and serum TSH level measured.

### RNA-Seq (DGE).

Mice (WT and THRB K146Q sumoylation mutant mice) at 3 months of age (*n* = 3/group) were euthanized, and the pituitary and hypothalamus were collected. Total RNA was isolated from both tissues. RNA-Seq was performed in the HiSeq 3000 system (Illumina), and data were analyzed by the UCLA Genetics Core. The RNA-Seq data for hypothalamus, pituitary, and thyroid have been deposited to NCBI Gene Expression Omnibus repository (GSE167105).

### Liquid chromatography–tandem mass spectrometry analysis of TH in the thyroid gland.

The method used was as previously described (68). In brief, mouse thyroid glands were dissected from WT and THRB K146Q mice (*n* = 18/genotype) and snap-frozen in liquid N_2_. Tissues were quickly digested using Pronase (MilliporeSigma). Thyroid hormones were extracted from the thyroid gland with a solid-phase extraction method and analyzed by liquid chromatography–tandem mass spectrometry.

### ChIP analysis of TSHβ gene in the pituitary.

Age- and sex-matched WT and mutant mice were divided into 3 groups: control (*n* = 13/group), hypothyroid (low iodine and 0.15% PTU diet for 3 weeks), and hyperthyroid (T3 10 μg/100 g body weight/d for 3 days). Serum TSH, T4, and T3 levels were measured after treatment. Pituitary and thyroid tissues were collected and analyzed using the ChIP Kit (MilliporeSigma), following the manufacturer’s instructions. In brief, tissues were first incubated with tissue chromatin stabilization buffer, and the DNA-protein complex was cross-linked using 1.3% formaldehyde (final concentration) for 10 minutes and stopped by the addition of glycine to a final concentration of 125 mM. Tissues were lysed in ChIP lysis buffer with complete protease inhibitors (Roche) and 2 mM PMSF. We optimized the sonication procedure to obtain 150 bp to 200 bp DNA fragments. The sonicated lysate was centrifuged for 15 minutes at 12,000*g* at 4°C and the supernatant was collected for IP. A 10% fraction of the supernatant was used as the input control. The antibodies used are listed in Table 3. Each antibody was pretested by IP using GH3 cell lysate. The same antibody lot number was utilized across multiple experiments to ensure uniformity of results. The negative control in the ChIP assay was rabbit IgG. Purified DNA from the ChIP assay was used in quantitative PCR, 2 μL DNA in 10 μL PCR reaction. For PCR primer sequences, see Table 4. The assay (PCR) data represent DNA-bound TFs with 10% input and labeled as relative enrichment.

### Statistics.

Comparisons between 2 independent groups were assessed with a 2-tailed Student’s *t* test. One-way ANOVA was used for comparing 1 factor in multiple groups. Two-way ANOVA was used in comparison of multiple groups. A *P* value of 0.05 or less is considered significant and is shown in the graphs. All data shown represent mean ± SD. GraphPad Prism version 9.02 was used for statistical analysis.

### Study approval.

All experimental procedures and animal use were approved by approved by the Animal Care Committee of VA Greater Los Angeles Healthcare Center and in compliance with federal guidelines.

## Author contributions

SK performed the majority of experiments. RK and KK analyzed TH species in thyroid tissue. JJ, FL, KA, and AM assisted in critical experiments. YL performed ChIP assays. YYL and GAB designed research, analyzed data, made all the figures, and cowrote the manuscript.

## Supplementary Material

Supplemental data

## Figures and Tables

**Table 1:**
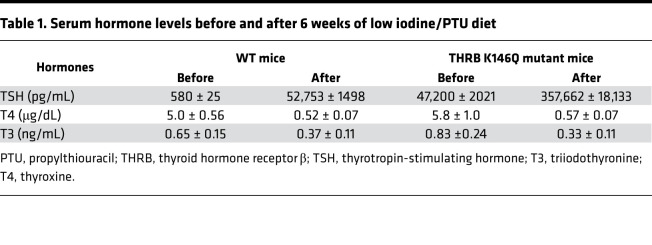
Serum hormone levels before and after 6 weeks of low iodine/PTU diet

**Table 2:**
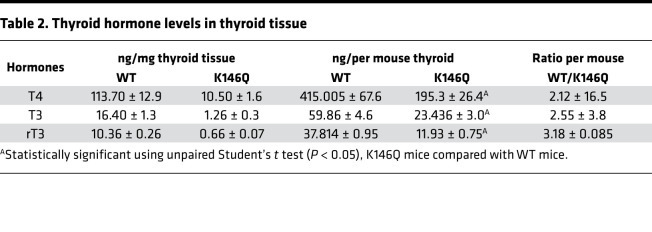
Thyroid hormone levels in thyroid tissue

**Table 3:**
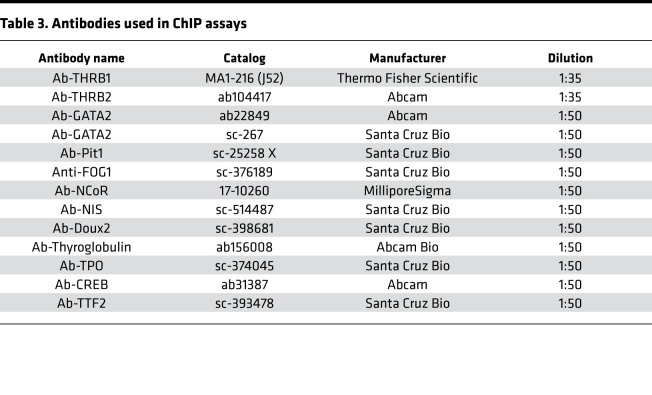
Antibodies used in ChIP assays

**Table 4:**
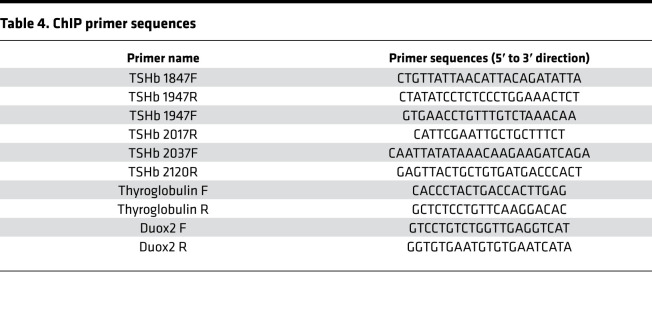
ChIP primer sequences

## Version 1 07/08/2021

In-Press Preview

## Version 2 08/23/2021

Electronic publication
