# Supplementary material for: Thyroid hormone receptor β sumoylation is required for thyrotropin regulation and thyroid hormone production
Source: JCI Insight. 2021 Aug 23;6(16):e149425. doi: 10.1172/jci.insight.149425 (PMC8410017; doi:10.1172/jci.insight.149425)
Supplement: Supplemental data [file jciinsight-6-149425-s225.pdf]

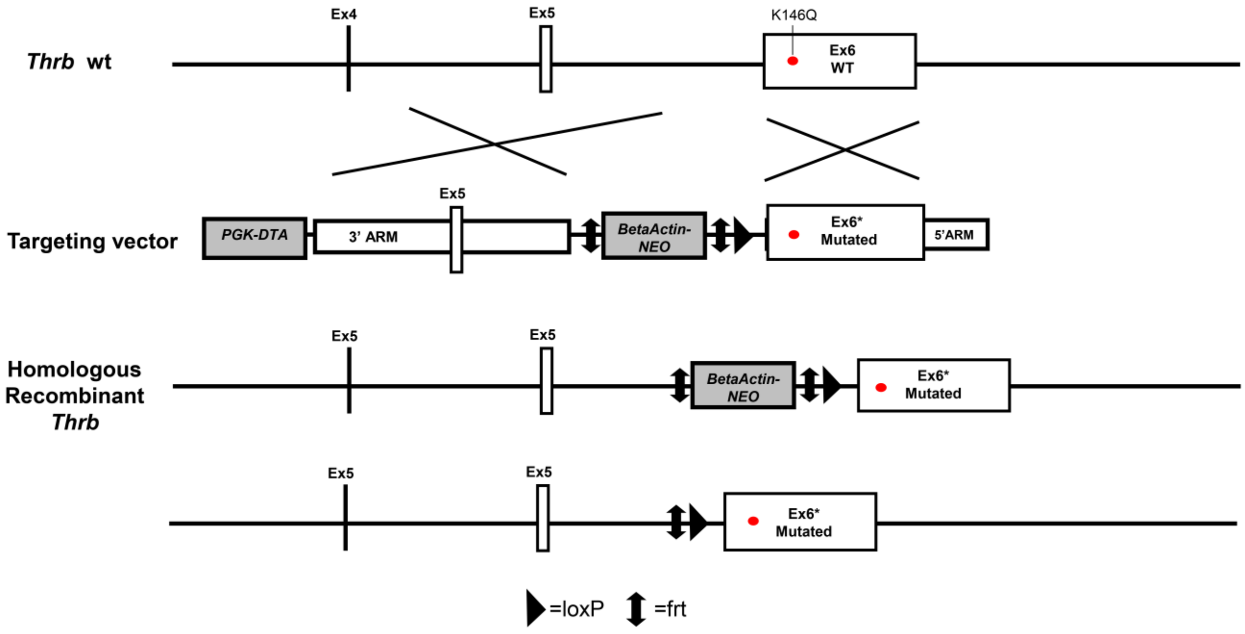

Supplemental Figure 1. Generation of a Knock-In allele, via a sequence replacement strategy, to mutate the *Thrb* gene. The targeting construct contained the K146Q mutation in exon 7, a 5.3 Kb 5' arm of homology (containing exon 6), a 5.3 Kb 3' arm of homology, a Diphtheria Toxin A (DTA) cassette, and a Neomycin (Neo) cassette flanked by frt sites for selective deletion. The Neo element allows for positive selection in embryonic stem (ES) cells, while the DTA element permits negative selection. After homologous recombination of the Knock-In construct, the Neo is excised after Flp-e administration. The homologous recombinant contains the K146Q mutation in the native *Thrb* allele.

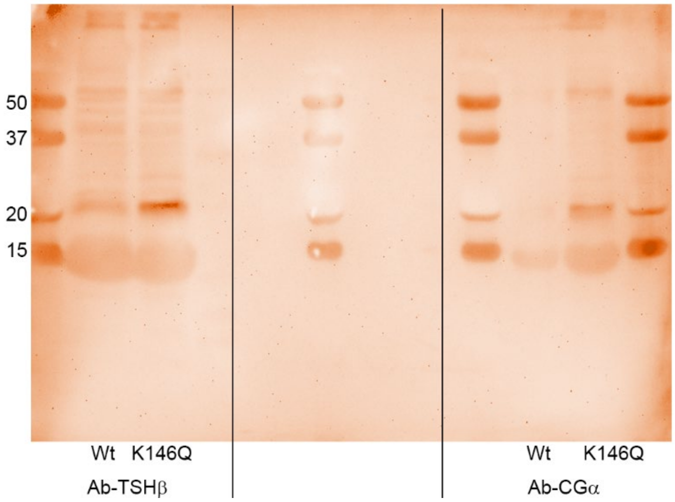

Supplemental Figure 2. Ponceau S stained PVDF membrane after protein transfer. Pituitary tissue was lysed in RIPA buffer with protease inhibitors. Protein (30μg) was loaded in each lane of a 14% SDS gel. After protein was transferred to the PVDF membrane, the membrane was stained with Ponceau S for 10 minutes and then tailored to the appropriate size for blotting with anti TSHβ and anti-CGα antibody, as is described in the material and methods.

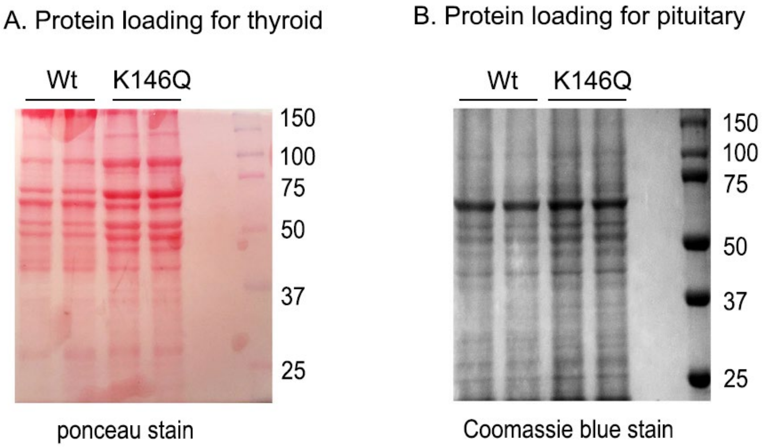

Supplemental Figure 3. Total protein loading. Thyroid and pituitary tissues were lysed in RIPA buffer with NEM and complete protease inhibitors. The solution was centrifuged at 12000 g, the supernatant was collected and protein concentration was determined. Protein (30ug) was loaded in each lane of a 10% SDS gel. After electrophoresis, proteins were transferred to a PVDF membrane. (A) Thyroid tissue, after transfer, PVDF membrane was stained with Ponceau S for 30 minutes and imaged. (B) Pituitary tissue, two gels were prepared with the same protein loading and run back-to-back, one gel for antibody detection and another for staining with Coomassie blue to determine total protein loading.

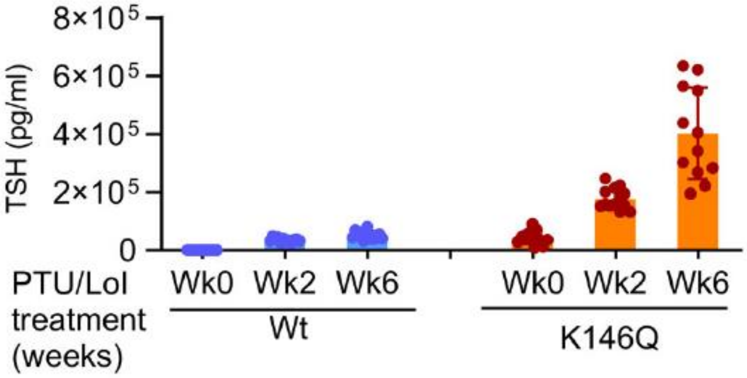

Supplemental Figure 4. TSH levels in Wt and K146Q mice after propylthiouracil/low iodine diet (PTU/LoI)-induced hypothyroidism. Mice were given low iodine diet supplemented with 0.15% propylthiouracil (PTU) (Envigo, TD.95125) for 6 weeks. Blood was drawn at baseline, week 0, and at weeks 2 and 6 of treatment. The plasma TSH level was analyzed by Elisa (Millipore Catalog # RTHYMAG-30K) and Luminex 200 Bioanalyzer. Two-way ANOVA analysis was performed and column factor, p <0.0001, indicating significant difference among the columns.

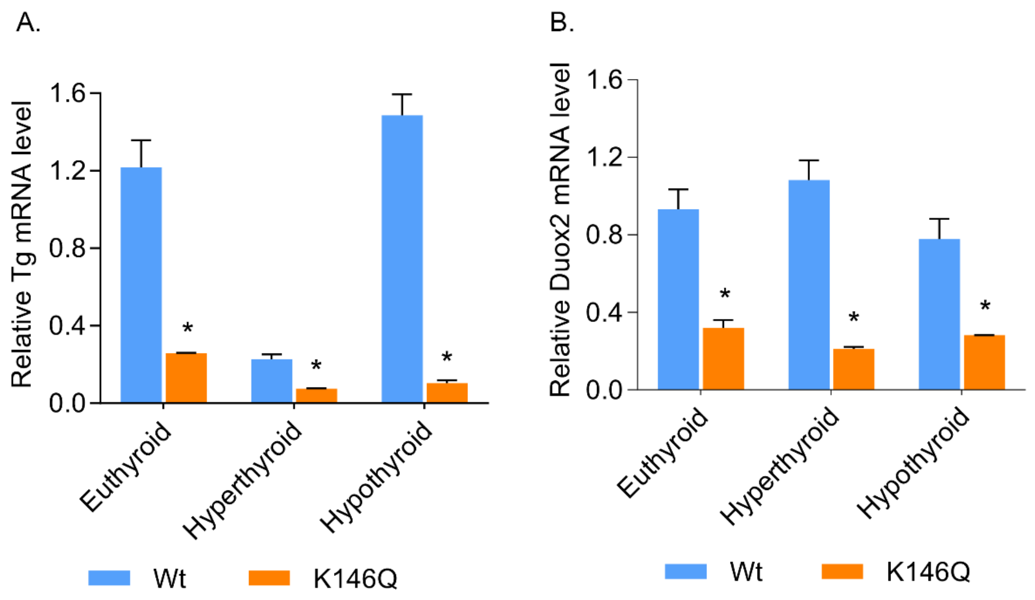

Supplemental Figure 5. RT-PCR quantification of thyroglobulin (*Tg*) and dual peroxidase (*Duox2*) mRNA expression. Wt and K146Q mice (n=3/ genotype) were treated with T3 (i.p. injection, 10 μg/100 g body weight) for 3 days to induce hyperthyroidism. Wt and K146Q mice (n=3/genotype) were fed a low iodine diet supplemented with 0.15% PTU for 3 weeks to induce hypothyroidism. The thyroid gland was then dissected and RNA was isolated and reverse transcribed using QuantiTec Reverse transcription kit (Qiange Inc). The cDNA was diluted 10-fold and 2 μl of the solution was used in RT-PCR with QuantiTect primer assay (Qiagen Inc) for Tg (ID-QT00116592) and Duox2 (ID-QT01764483). Three housekeeping genes were used for the normalization of *Tg* and *Duox2* mRNA levels, *Beta 2 macroglobulin* (B2M, D-QT01149547), *18S rRNA* (Rn18s, ID-QT02448075) and *Hypoxanthine-guanine phosphoribosyltransferase* (Hprt, ID-QT00166768). *Beta-glucuronidase* (GUSB, ID - QT00176715). Statistical comparison of mRNA levels in Wt vs K146Q mice was performed using paired Student's *t* test. (* $p<0.05$).
